# Editorial

**Published:** 1846-09

**Authors:** 


### THE MEDICAL EXAMINER.

PHILADELPHIA, SEPTEMBER, 1846.

### ANNUAL CIRCULARS OF MEDICAL COLLEGES AND CLINICAL MEDICINE.

Medical teaching, like the other avocations of men, naturally adapts itself to the spirit of the age. Even in speculative Germany, in the present day, a utilitarian spirit begins to prevail. Her physicians and philosophers are less enamoured with hypotheses and baseless theories than formerly, and have set themselves down, many of them, as patient investigators of nature, content to witness and record phenomena as they are observed, without too hastily seeking to connect them by uncertain or fancied resemblances. In France, the same tendency exists, and may be seen, almost in the extreme, amongst the followers of Louis. In Great Britain and the United States, a more middle path has generally been pursued. The physicians of this country, in particular, separated by the Atlantic, and having few entangling alliances with those of Europe, have been less dazzled by the meteors that at various times have flitted across the horizon of the continent, and, with the quiet contentment which characterizes the unambitious, have received whatever of good was presented to them by their more industrious neighbours, without much caring for the source from whence it came. But a change has come over us, and the impertinent question will hardly again be asked, "who reads an American book?" etc. Books that even foreign critics applaud, are written; discoveries are made; experiments, original in their conception and startling in their results, are instituted; and surgical operations, ingenious, novel and successful, are performed. These things denote that the age of absolute dependence has passed, and that in literature and science, as well as in government, we shall henceforth hold a place among the nations of the earth. In medical teaching, the same advance to national manhood may be seen. Formerly, in the few colleges for granting instruction in medicine that existed, some of the more important branches were totally neglected. It is but a few years since attendance upon lectures on Midwifery was deemed necessary for graduation, in the first school in the country.

The institutes and forensic medicine, as is still the case in too many schools, were unknown and untaught to the students. Even the didactic lectures that were given on what were considered as the necessary branches of medical education were seldom delivered orally, and were almost never accompanied by diagrams or other means of demonstration!

In those days, no one was supposed to have a complete medical education who had not visited Edinburgh, or some other European seat of medical learning. Now, it is not pretended that such a tour is necessary, except for the purpose of witnessing the economy of the larger hospitals, and more especially by those who desire to devote themselves to what are called specialties. For the purposes of the latter, the great number of cases collected in the larger Hospitals of London and Paris, afford unusual means; but, for the general purposes of clinical study, the various towns of the United States where medical schools exist, and particularly the Atlantic cities, afford the the desired facilities to an extent far greater than most students are willing to avail themselves of.

The practice of American students, rendered necessary in too many instances perhaps by their limited pecuniary means, of spending no more time in our large cities than is occupied by the public lectures, necessarily precludes them from witnessing much clinical practice. This is greatly to be deplored; and but for the plan, which has sprung up as the consequence, of bringing patients into the amphitheatres of the colleges to be prescribed for and operated on in the presence of the students, many of them would, as formerly, go into practice without the least instruction of the kind. By this plan, the principles of clinical medicine can be taught, and to some extent the practice can be witnessed, even in villages. In the large cities, from the great number of poor patients, by proper exertion on the part of those concerned, it is in the power of the teachers of the colleges to exhibit to their classes, during a course, nearly every form of disease (except a very few of the more acute and therefore least obscure) which flesh is heir to, as well as most of the surgical operations occurring in private practice. In this way a large number are taught a great amount of useful knowledge who never would have been taught it in any other way. The serious farce of hundreds racing round the wards of an hospital after a prescriber whom few of them can approach, to view patients whose very faces they cannot see, and to listen to the remarks of a teacher whose voice they scarcely can distinguish in the general confusion that prevails, is avoided; and every one can see what is attempted to be shown, and hear what is said for his edification. No one will pretend that this is a perfect system of clinical teaching: certainly a better could be devised for the instruction of a small number of pupils in the wards of an hospital; but in this way those who are qualified and desirous of learning may be taught a great deal that is not merely useful but highly important. They can be taught how to examine a patient—the value of symptoms, and their relation to the pathology of the case—the bearing of the general principles of pathology on the particular case—the therapeutical indications, and the means adapted to their fulfilment. All this can be taught clearly and intelligibly, if the instructor is competent to the task, to hundreds of students as readily as to ten. It is true that in this way the students do not feel the patient's pulse, nor examine his secretions and excretions; they do not auscultate his chest nor percuss his abdomen—but all this is done for them and in their presence by the teacher, and the facts are announced to them, with the proper comments. To object to this mode of teaching is to object to half a loaf of bread when you cannot get a whole one—it is to object to the education of a student of surgery because, although he may have been taught anatomy and the principles of surgery, he has not himself during his clinical attendance performed amputation, trephining, lithotomy, and every other operation within the range of the art—it is to object to the physician as deficient in a knowledge of his profession, because, although profoundly acquainted with the principles of pathology and therapeutics, he has not seen and himself treated the plague and yellow fever! Those who advocate this "new-fashioned system," as it has been called, do it not on the ground of its being a full or complete mode, but as the best under existing circumstances--as the best way in their power of teaching a large class of young men how to study a case and prescribe for it, when they come to sit by the bed side themselves. It lies not in the way of fuller instruction, but is an introduction to it. That such are the views of those who preside over the medical schools of the country is apparent from the circulars put forth by many of them the present year. The practice of giving clinical instruction in this mode has prevailed in some of the principal colleges for years, and now it obtains generally. In many of the annual circulars, not only is the fact announced, but a list of the cases treated during a previous session is appended, to enable the profession to judge of the extent and variety of the cases witnessed by the class.

What does it concern a student to be told that throughout the thousand miles of streets, lanes and alleys that divide and in a hundred ways subdivide a large city, forty, fifty or a hundred thousand persons are prescribed for in the course of the year? The patients of the public dispensaries and other public charities, scattered over so large a space, each at his own home, are, as alluded to under another head, as inaccessible to a student whose every hour in the day is occupied with the lectures in a college, as if they were in the Celestial Empire. He cannot go to them, and if he is to see them at all they *must be brought to him.*

---

### OHIO MEDICAL CONVENTION.

By the published "Proceedings of the Ohio Medical Convention," of the present year, we learn that it assembled at Columbus on the 12th of May last. The following gentlemen were proposed by the nominating committee for the several offices, and duly appointed, viz:

| | |
|---|---|
| President, | Dr. A. H. Baker, of Preble county. |
| 1st Vice President, | M. Z. Kreider, Fairfield county. " |
| 2nd " " | J. B. Thompson, Franklin " |
| 3rd " " | William Judkins, Hamilton " |
| 4th " " | ——— Price, Licking " |
| 5th " " | A. M. Thompson, Knox " |
| 6th " " | ——— Hildreth, Muskingam " |

Drs. J. F. Hibberd and S. M. Smith were appointed Secretaries.

The Convention appears to have been fully attended—several papers were read, a code of Ethics adopted, and various other matters of interest to the profession considered.

Some of the papers read on the occasion we shall transfer to our pages in a future number.

Among the resolutions, the following proposed by Dr. Hibberd was adopted: "Resolved, That Professor M. B. Wright, of the Medical College of Ohio, Prof. Samuel St. John, of the Medical College at Cleveland, and Prof. John Butterfield, of the Medical College at Willoughby, be appointed delegates to represent this Convention in the National Medical Convention, to assemble in Philadelphia in May, 1847, and that each have power to appoint an alternate."

---

### CATALOGUES AND CIRCULARS OF MEDICAL COLLEGES.

We have received a number of these annuals, some of which we have unfortunately mislaid. The following are the most recently received.

*Annual Catalogue of the Officers and Students of the College of Physicians and Surgeons, of the City of New York.*—The number of regular Matriculants for the Session of 1845-6, was 218 ; being 25 more than the preceding session—a greater number, we are informed, than in any previous session of the Institution.

*Catalogue of the Trustees, Faculty, and Students of the Medical College of the state of South Carolina—Session of* 1845-6.—"The class in attendance amounted to two hundred and ten students," from five States.

*Circular of the Medical Department of the Columbian College, Washington, D. C.* 1846.—Number of the last class not stated.

We are gratified to find by these circulars, that the respective institutions are in a healthy condition, and bid fair to continue to send forth able and well instructed physicians.

All have adopted the plan of giving clinical in connection with didactic instruction in the college buildings, and the two last mentioned append to their circulars a list of patients treated during the last session.

*Seventh Annual Announcement of the Baltimore College of Dental Surgeons.*—This publication announces the gratifying fact, that a College for teaching Dentistry on scientific principles *can* be sustained in the United States. In no country is the art better taught or better practised, and those who aspire to become members of the fraternity hereafter, must, nolens volens, seek to accomplish themselves, or be content to rank among the quacks of the profession.

---

### M. FLOURENS.

By the *London Lancet* of the first of August, we learn that this distinguished *Savan* has been created a Peer of France. The Peerage will undoubtedly be honored by this association, but what honor can it confer on the name of Flourens ?

